# English Hospitals and English Diplomas

**Published:** 1909-02-13

**Authors:** 


### The Hospital

A Journal of

The Medical Sciences and Hospital Administration.

New Series. No. 102, Vol. IV. [No. 1174, Vol. XLV.] SATURDAY, FEBRUARY 13, 1909.

### ENGLISH HOSPITALS AND ENGLISH DIPLOMAS.

Although we deprecate those unhappy personal disputes and local or racial controversies which agitate from time to time the waters of medical politics, and would prefer not only to maintain a neutral attitude ourselves, but also to avoid any expression of opinion which might possibly be construed into a sign of prejudice or bias, there are yet occasions when to keep silence may be to play traitor to both sides. Thus, a total lack of reference in the editorial columns of a medical journal to those acute stages, which periodically arise in the course of old-standing grievances and disputes, may be prompted by the best of intentions towards both parties; but it is, nevertheless, liable to misinterpretations of the most varied kind, and only too often fails hopelessly in its object of conciliation. Moreover, in such matters as that which we now have in mind—namely, the exclusion of candidates not holding the higher diplomas of the London College of Physicians or the English College of Surgeons from most of the London and many of the provincial hospital posts—a perfectly neutral attitude is almost impossible, and its assumption savours of hypocrisy. We may, indeed, feel that there is much to be said on both sides, and we may earnestly desire to maintain a judicial position; but, unless we would deserve the reproach of indifference, the balance of our judgment must come to rest on one side of the beam or the other. Therefore, since silence is certain to be construed in some quarters into passive partisanship, it seems wiser to state our views on such questions, firmly yet temperately, as soon as we have had time to consider them in all their present bearings. This course may lead us to grate against the sentiments and convictions of a certain section of our readers; but it will at least clear the controversy of some of its meaner features, and prevent misunderstanding.

In the first place we would distinguish between the words hardship and injustice. Much of the bitterness and protraction of these controversies is due to the inaccurate use of words and phrases. This inaccuracy—often reckless, sometimes wantonly reckless—is, we believe, chiefly due to ignorance, or (in more polite terms) to lack of appreciation of the precise value of words. That the English vocabulary is twisted in the present instance into a deliberate means of provocation we do not believe. Now we must admit at once that the restriction of candidature for honorary posts in many English hospitals to those possessing the higher diplomas of the London colleges occasionally inflicts hardships upon Scottish and Irish practitioners of the very highest skill and attainments, who are in all other respects admirably qualified for the work. We sympathise, therefore, to that extent with the object which lately brought together in London so many eminent Irish medical men, under the auspices of the Irish Medical Schools' and Graduates' Association and the Scottish Medical Diplomates' Association. But we think that, in the exposition of their grievance and in the advocacy of remedial measures, some of those who then spoke, and some who have since written in the journal especially devoted to their interests, have used stronger words than are expedient. Racial, religious and professional controversies all require very temperate language. Such words as monopoly, injustice, exclusion, favouritism, and so forth should be used with caution; for their meanings are flexible, and vary widely according to the attitude of the speaker and the listener. Unless discharged with much circumspection, they are apt to hit too hard, or to hit the wrong object, or even to recoil upon the marksman.

We think the governors of the pilloried London and provincial hospitals should be credited with the wish to secure, according to their lights, the best available men for their posts, and to obtain them in the simplest and most expeditious fashion. Let us take the case of the higher surgical posts. Here the governors, by their conditions of candidature, display their acquiescence in the very widespread belief that the Fellowship of the Royal College of Surgeons of England is the premier surgical diploma of the world, and stands for the high-water mark of surgical knowledge and ability—so far as these can be gauged by curriculum and examination. This belief implies no slur upon the skill or learning of the many distinguished surgeons who do not possess this diploma. But it at least extenuates the present method of reducing to wieldly proportions the candidature for London surgical posts. As a preliminary the governors require a certain hall-mark; the local hall-mark, it is true, but the best one they know, and one with a world-wide currency value. Other gold may be as good or better, but this hall-mark ensures a certain definite minimum of attainment. The whole thing is open and above board; there is no general invitation to all comers, followed by a private system of local favouritism. Irishmen and Scotsmen having the required diplomas adorn the staffs of many London and provincial hospitals, and are welcomed by governors and colleagues alike. In this respect London is far more generous and infinitely less exclusive than the four great cities of Scotland and Ireland.

If all monopolies are wrong, then the English custom is wrong. It is certainly undemocratic and it may be inhospitable. It certainly inflicts some hardship (but not from any personal or racial motive) upon a few individuals. It compels others to extend or modify their studies and qualifications; while in the case of physicians it enforces certain restrictions of practice which are peculiar to Fellows or Members of the London College. But is it wholly unjust and indefensible? In any case it is not a "put up job," as is alleged, between "antiquated and irresponsible" governing bodies, qualifying corporations, and medical staffs, but an honest means of selection which has worked well in the past; and whatever its faults it scarcely seems to call for such drastic treatment as abolition by the State, or interference on the part of the General Medical Council.

